# Contamination identification, source apportionment and health risk assessment of trace elements at different fractions of atmospheric particles at iron and steelmaking areas in China

**DOI:** 10.1371/journal.pone.0230983

**Published:** 2020-04-02

**Authors:** Xiaoteng Zhou, Vladimir Strezov, Yijiao Jiang, Xiaoxia Yang, Tao Kan, Tim Evans

**Affiliations:** 1 ARC Research Hub for Computational Particle Technology, Macquarie University, Sydney, New South Wales, Australia; 2 Department of Earth and Environmental Sciences, Macquarie University, Sydney, New South Wales, Australia; 3 School of Engineering, Macquarie University, Sydney, New South Wales, Australia; Institute for Advanced Sustainability Studies, GERMANY

## Abstract

China has the largest share of global iron and steel production, which is considered to play a significant contribution to air pollution. This study aims to investigate trace element contamination at different fractions of particulate matter (PM) at industrial areas in China. Three PM fractions, PM_2.1–9.0_, PM_1.1–2.1_ and PM_1.1_, were collected from areas surrounding iron and steelmaking plants at Kunming, Wuhan, Nanjing and Ningbo in China. Multiple trace elements and their bioavailability, as well as Pb isotopic compositions, were analysed for identification of contaminants, health risk assessment and source apportionment. Results showed that PM particles in the sites near industrial areas were associated with a range of toxic trace elements, specifically As, Cr(VI), Cd and Mn, and posed significant health risks to humans. The isotopic Pb compositions identified that coal and high temperature metallurgical processes in the steelmaking process were the dominant contributors to local air pollution in these sites. In addition to iron and steelmaking activities, traffic emissions and remote pollution also played a contributing role in PM contamination, confirmed by the differences of Pb isotopic compositions at each PM fraction and statistical results from Preference Ranking Organization Method for Enrichment Evaluations (PROMETHEE) and Geometrical Analysis for Interactive Aid (GAIA). The results presented in this study provide a comprehensive understanding of PM emissions at iron and steelmaking areas, which helps to guide subsequent updates of air pollution control guidelines to efficiently minimise environmental footprint and ensure long term sustainability of the industries.

## Introduction

Iron and steel manufacturing is a staple of the world’s industrial economy with a total estimated worth of $900 billion per year [1]. Almost everything used today, such as housing, transport, energy production, food and water supply, is either made of steel or manufactured by steel equipment [2]. Although its production increasingly improves our daily life, its negative impact on the environment cannot be ignored [3–5]. According to the World Health Organization [6], iron and steel manufacturing is a significant contributor to air pollution, especially in developing countries which have an increasing demand for steel in the domains of industrialisation and modernisation [7, 8].

China has witnessed a significant growth in steel production since 1996 [9]. Its total crude steel production was 928 million tonnes in 2018, contributing to 51.3% of the global crude steel production [10]. Airborne dust emissions in China were estimated at 8–17 million tonnes per year [11], with 27% resulting from iron and steelmaking industries [12]. The subsequent damage to the environment is reflected by the statistical analysis provided by China National Environmental Monitoring Centre, whereby the average PM_2.5_ concentration produced by the top 20 cities in Chinese steel production list was 28% above the national average data [13]. For this reason, it is essential to better understand the airborne PM concentration and chemistry, and its potential health risks at iron and steelmaking areas in China in order to establish efficient control measures.

PM particles emitted from iron and steelmaking industries have been evidenced to include a range of toxic trace elements, posing a significant health risk [3, 14, 15]. Previous studies have found that the distribution of toxic trace elements is associated with the PM size [16–18]. For example, the trace elements As, Cd, Cr, Ni and Pb, which present significant health risks at low concentrations, tend to be accumulated in fine particles [19, 20]. However, the trace elements Fe and Zn, associated with negative health effects at high concentrations, were found accumulated in coarse PM particles [21]. Given the toxicity of trace elements varies, it is necessary to consider toxic factors for each individual element when investigating contamination levels at different particle sizes.

Nikolic [22] and Ilić [23] defined weight coefficients for key trace elements using the Preference Ranking Organization Method for Enrichment Evaluations (PROMETHEE) and Geometrical Analysis for Interactive Aid (GAIA) analysis to identify the contamination of smelting emissions in Europe. PROMETHEE and GAIA multivariate analysis methods have been widely used in the past to indicate the most preferred objects for decision making. For instance, they were used to rank contamination levels as a result of elevated trace elements at different sizes of sediment particles in Australia [24–27]. However, to date, PROMETHEE and GAIA multivariate analysis methods, which are based on toxic coefficients of trace elements, have not yet been used to estimate air quality across different atmospheric fractions.

The toxicity of an individual trace element is dependent on its state [28]. For example, airborne Cr has two primary forms of Cr(III) and Cr(VI) [29]. Trivalent Cr is considered to be an essential nutrient, while hexavalent Cr has been evidenced to be associated with an increased risk of lung and nasal cancer [30] and is classified by the U.S. Environmental Protection Agency [31] as ‘Group A–Carcinogenic to Humans’. Cr and its compounds are widely used by the metallurgical industry, which is the dominant source of airborne Cr emissions into the environment [30]. However, previous studies used 1/6^th^ of the total Cr concentrations as the hexavalent Cr for health risk assessment [32–36], and limited studies investigated the behaviour of extracted Cr(VI) concentrations in different atmospheric fractions coupled with assessment of their corresponding cancer risks at iron and steelmaking industrial areas [37].

PM particles are not only emitted from industrial sources but are also contributed by local traffic emissions or transported from remote sources as a result of atmospheric movement [6]. Pb isotopic composition analysis is an established tool to identify the contamination sources of trace elements in PM_10_ and PM_2.5_ emitted from coal burning, traffic emissions and metallurgical dust [38].

This study aims to investigate chemical composition and source apportionment of PM particles near iron and steelmaking industrial areas in China. The collected PM samples at different sizes were subjected to a range of chemical analyses, including trace element concentrations and bioavailability analyses as well as Cr(VI) extraction and Pb isotopic composition determination. The multicriteria analysis methods PROMETHEE and GAIA were applied to determine contamination levels across different atmospheric fractions. The potential health risks were assessed based on the extracted Cr(VI) concentrations coupled with other multiple trace elements near iron and steelmaking areas. The study further provides a Pb isotopic composition analysis to verify the influence of wind on contamination of atmospheric particles near ironmaking industrial sites. The results presented in this study are of significance to better understand and control PM emissions in these industrial areas.

## 2 Materials and methods

### 2.1 Sampling information

**Fig 1:**
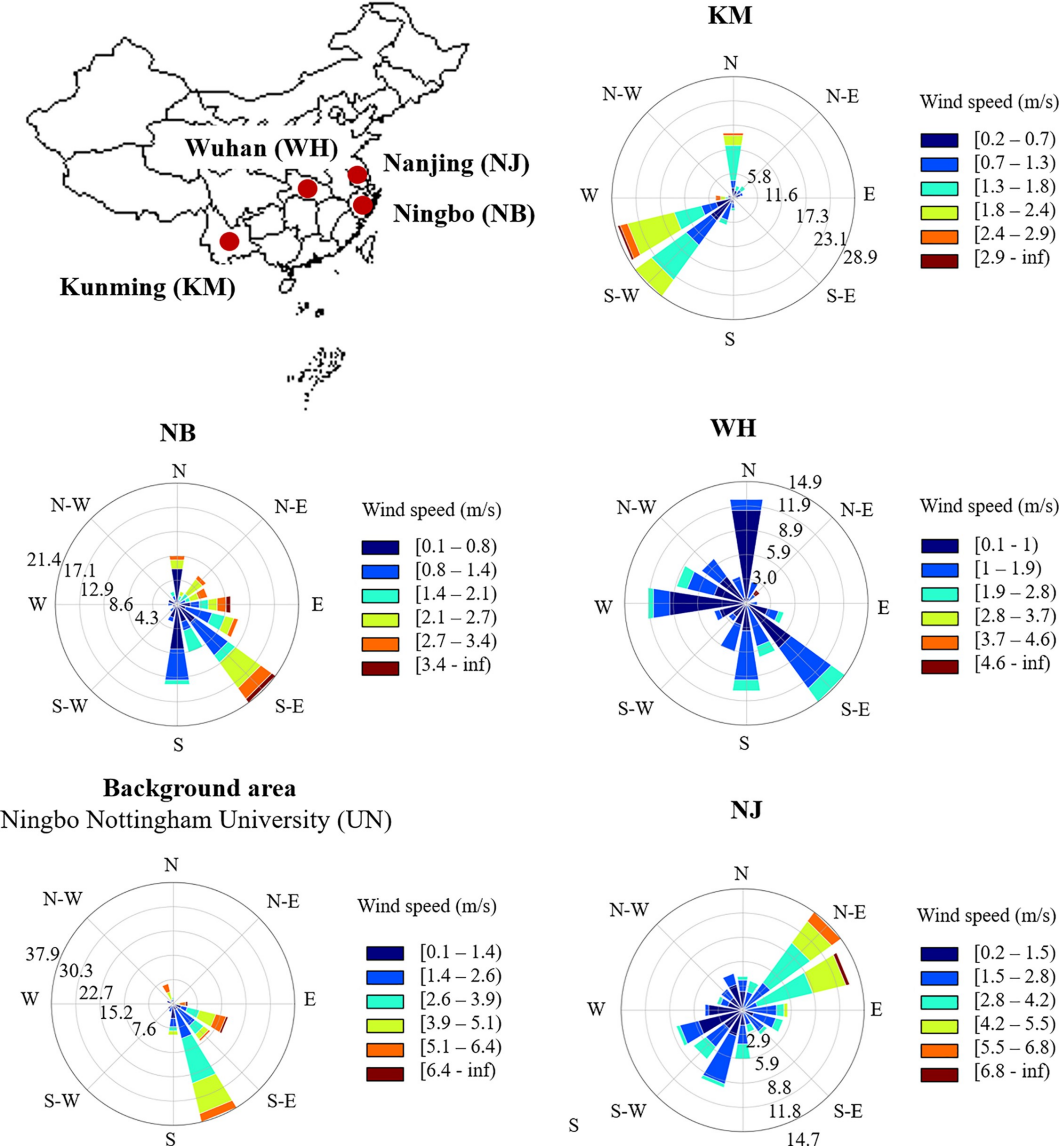
Sampling locations and wind information at four steel industrial areas in Kunming (KM), Wuhan (WH), Nanjing (NJ) and Ningbo (NB) and the background area located at Ningbo Nottingham University (UN). The industrial plant at each sampling site and the UN campus were outlined with green lines and red star symbols to highlight the sampling locations. The sampling locations at four industrial areas were within < 1 km away from the steel plants.

Four steel industrial areas investigated in this study were located at Kunming (KM) (24.8801° N, 102.8329° E), Wuhan (WH) (30.5928° N, 114.3055° E), Nanjing (NJ) (32.0603° N, 118.7969° E) and Ningbo (NB) (29.8683° N, 121.5440° E) (Fig 1). In addition, one background site at Ningbo Nottingham University (UN) was selected in this study (Fig 1). PM samples at five sampling sites were collected every 24 hours over a period of five days with similar environmental and meteorological conditions during April–July 2017. The temperature at KM, WH, NJ, NB and UN ranged from 10–26°C, 18–33°C, 19–34°C, 17–28°C and 18–28°C, respectively, during the sampling period. The total precipitation was zero at KM, NJ and UN, while it was 5.6 mm at WH and 67 mm at NB which is a coastal city. The information of wind direction and speed at each sampling site is shown in Fig 1. The HYSPLIT backward trajectory at each sampling site was also provided to describe the origin of air masses and potential emission sources from remote areas during the sampling period (S1 Fig).

The steel mill at KM which was built in 1939 was the oldest industry among the four selected plants in this study, while the plant at NB was most recently established in 2003 and equipped with advanced particle capture instruments. The plants at WH and NJ were both build in 1958, but WH steel mill had the largest annual production of iron and steel at 20 million tonnes. Further information for the plant conditions of each industrial sampling location is detailed in Table 1.

**Table 1:** Industrial information on iron and steel plants at Kunming (KM), Wuhan (WH), Nanjing (NJ) and Ningbo (NB) in China.

		KM	WH	NJ	NB
**Plants**	**Established year**	1939	1958	1958	2003
	**Iron and steel production per year (million tons)**	7	20	9	4
	No. of blast furnace (BF)*	1	8	5	2
	No. of blast oxygen furnace (BOF)*	3	10	6	3
	No. of electric arc furnace (EAF)*	0	0	1	0
	**Air pollution control measurements**	1) Limestone gypsum flue gas desulfurization; 2) Electrostatic precipitators; 3) Bag filters;	1) Limestone gypsum flue gas desulfurization; 2) Electrostatic precipitators; 3) Bag filters; 4) Wet scrubber;	1) Limestone gypsum flue gas desulfurization; 2) Bag filters; 3) Wet scrubbers; 4) Long bag type pulse filters;	1) Electrostatic precipitators; 2) Bag filters; 3) Wet scrubbers;
	**Recirculation system**	No	No	No	Flue gas recirculation
**Sampling locations of plants**		Downwind	Downwind	Downwind	Upwind

*Facility information was obtained from Yang [39].

### 2.2 Sample collection

Non-viable Andersen cascade impactor with 8 aluminium state plates (Model 20–800, Tisch Environmental) was installed at local meteorological sites or on the roof of high buildings to collect atmospheric particles at the five sampling sites shown in Fig 1. The 50% cut off diameters (D_50_) of the Andersen sampler stages were 9.0, 5.8, 4.7, 3.3, 2.1, 1.1, 0.65 and <0.43 μm. Atmospheric particle samples (n = 200, eight samples per site per day) with different size fractions were collected from each sampling site for 24 h loading at a flow of 28.3 L/min. All the samples were stored at 4°C until analysis.

### 2.3 Laboratory analysis

A five 24-hour sampling protocol was conducted for each sampling sites. Filters from two days of sampling were subjected for Cr(VI) analysis, filters from the third day were used for bioavailability analysis and the last two-day samples were used for multiple trace element analysis and Pb isotopic composition analysis. All the samples were analysed using an inductively coupled plasma optical emission spectrometer (ICP-OES, Varian 730-ES) and an inductively coupled plasma mass spectrometer (ICP-MS, Agilent 7900) at the Australian National Measurement Institute, Sydney, Australia.

#### 2.3.1 Cr(VI) analysis

Air filter samples with PM_2.1–9.0_, PM_1.1–2.1_, and PM_1.1_ size fractions across five sampling sites over two days were selected for Cr(VI) analysis. Samples were digested using a Na_2_CO_3_/NaOH solution and heated at 100°C for three hours to extract Cr(VI) and stabilize against reduction to Cr(III). The solution was diluted 10 times using 1 mL of supernatant adding 0.3 mL HNO_3_ (15.6 M) and making up to 10 mL with Milli Q water (18.2 MΩ·cm) prior to ICP-MS analysis. A water insoluble Cr(VI) blank spike (0.201 mg/L PbCrO_4_) and a Cr(III) blank spike (0.98 mg/L CrCl_3_·6H_2_O) were used to assess the accuracy of the extraction procedure for Cr(VI). The recovery rates of spike samples ranged from 91 to 109%.

#### 2.3.2 Bioavailability analysis

In order to assess ingestion health risks caused by trace element concentrations at different atmospheric particle sizes, the bioavailability analysis for trace elements was carried out in this study. All the air filters were folded and placed into 12 mL of graduated polypropylene centrifuge tubes. 10 mL of HCl (1 M) was added before tumbling for 1 hour. All the samples were diluted twice prior to ICP-MS analysis. Trace element concentrations for Al, As, Ba, Bi, Cd, Cr, Cu, Fe, La, Mn, Pb, Sb, Sr, V and Zn were determined for their bioavailable concentrations via ingestion exposure. Their procedural blanks were below the Limit of Reporting (LOR) of < 0.01 mg/kg and the recovery rates ranged from 96 to 116%.

#### 2.3.3 Multiple trace element analysis

The filter samples were digested using HCl and HNO_3_ (1:3, respectively, 8 ml) for 2 h. Each digested sample was topped up to 40 mL with Milli-Q water. Samples were diluted twice prior to analysis for Al, Ca, K, Mg and Na concentrations on an ICP-OES instrument. Concentrations of As, Ba, Bi, Cd, Ce, Cr, Cu, Fe, La, Mn, Pb, Sb, Sr, Ti, V, W, Zn and Zr were analysed using an ICP-MS instrument. Each sample batch (n = 20) contained a filter blank and duplicate, blank spike, blank matrix and matrix spikes. Procedural blanks were below the LOR of < 0.05 mg/kg for Al, Ca, K, Mg and Na, and < 0.01 mg/kg for As, Ba, Bi, Cd, Ce, Cr, Cu, Fe, La, Mn, Pb, Sb, Sr, Ti, V, W, Zn and Zr. Recovery rates for Al, Ca, K, Mg and Na ranged between 93−105% for all samples. Recovery rates for As, Ba, Bi, Cd, Ce, Cr, Cu, Fe, La, Mn, Pb, Sb, Sr, Ti, V, W, Zn and Zr for air filters were 95−110%.

#### 2.3.4 Pb isotopic composition analysis

Atmospheric samples collected from KM (PM_2.1–9.0_, PM_1.1–2.1_, PM_1.1_), WH (PM_2.1–9.0_, PM_1.1–2.1_, PM_1.1_), NJ (PM_2.1–9.0_, PM_1.1–2.1_, PM_1.1_), NB (PM_1.1_) and UN (PM_1.1_) were subjected to Pb isotopic composition analysis (^204^Pb/^207^Pb, ^206^Pb/^207^Pb, ^208^Pb/^207^Pb) after sample volumes were optimized on the basis of their Pb concentrations. The National Institute of Standards and Technology SRM981 was used to correct mass fractionations, and certified values of SRM981 for ^204^Pb/^207^Pb, ^206^Pb/^207^Pb and ^208^Pb/^207^Pb are 0.0646 ± 0.000047, 1.0933 ± 0.00039, and 2.3704 ± 0.0012, respectively. The mean RSDs for sample analysis ^204^Pb/^207^Pb, ^206^Pb/^207^Pb and ^208^Pb/^207^Pb were 0.43%, 0.26% and 0.16%, respectively.

### 2.4 Data analysis

#### 2.4.1 PROMETHEE and GAIA

PROMETHEE is a non-parametric method capable of ranking objects from the most preferred to the least preferred. In this study, PROMETHEE was applied to indicate the most polluted atmospheric particles and the most polluted sampling sites based on multiple variables.

The V shape preference function was selected for all the trace elements based on the maximum values as preference thresholds for each variable. The weights for the trace elements were referred to the toxicity points of each trace element outlined in Substance Priority List of Agency for Toxic Substances and Disease Registry (ATSDR 2017). The details of modelling parameters, including weight coefficients, preference functions, thresholds and trace element concentrations, were compiled in the S1 Table for the PM fractions at 2.1–9.0 μm, 1.1–2.1 μm and < 1.1 μm.

GAIA is a descriptive complement to the PROMETHEE rankings. It provides more profound insight into the relations between samples ranked with PROMETHEE and information on the variables responsible for the ranking. In a GAIA biplot, variables are considered to have positive correlations if their projected vectors form acute angles, negative correlations if they form obtuse angles, and no correlation if they are orthogonal [40, 41].

#### 2.4.2 Health risks

The exposure concentration (EC_inhalation_) and average daily dose (ADD_ingestion_) at three fractions of PM_2.1–9.0_, PM_1.1–2.1_ and PM_1.1_ were calculated according to Eqs 1 and 2.

$${EC}_{inhalation}=C_{inhalation}\times \frac{ET\times EF\times ED}{AT\times 24}\times CF$$ Eq 1

$${ADD}_{ingestion}=C_{ingestion}\times \frac{IngR\times EF\times ED}{BW\times AT}\times CF$$ Eq 2

Where C_inhalation_ refers to the total concentrations of trace elements for the PM fractions PM_2.1–9.0_, PM_1.1–2.1_ and PM_1.1_ (ng/m^3^, S1 Table); C_ingestion_ refers to trace element concentrations extracted from the bioavailability analysis (mg/kg, S2 Table); ET is exposure time (ET = 24 hours/day in this study); EF is exposure frequency (EF = 365 days/year in this study); ED is exposure duration (ED = 24 years in this study); AT is the average time (AT = ED × 365 days for non-carcinogens, AT = 70 × 365 days for carcinogens); IngR is the ingestion rate of 100 mg/day for adults [42]; BW is the average body weight (BW = 66.1 kg for male and BW = 57.8 for female) [43]; CF is the conversion factor of 10^−6^.

The health risks were characterised by non-carcinogenic and carcinogenic effects using equations of hazard quotient (HQ) and cancer risk (CR), respectively (Eqs 3 and 4).

$$HQ=EC/RfCi{=ADD}_{ingestion}/RfDo$$ Eq 3

$$CR=EC\times IUR={ADD}_{ingestion}\times SFo$$ Eq 4

RfCi is chronic inhalation reference concentration (mg/m^3^); RfDo is chronic oral reference dose (mg/kg·day); IUR is inhalation unit risk (μg/m^3^)^-1^; SFo is oral slope factor (mg/kg·day)^-1^. All the parameters used in Eqs 3 and 4 were obtained from U.S. EPA [44].

When the values of HQ for individual trace elements or the sum of HQ (∑HQ) are higher than one, then a chance exists for non-carcinogenic effects due to inhalation or ingestion exposure [45]. The tolerable value of CR at 10^−6^ means that the risk of developing cancer over a human lifetime (70 years) is one out of 1,000,000 people.

## 3 Results

### 3.1 PROMETHEE and GAIA

The PROMETHEE analysis was used to determine the most detrimental particle fraction and the site at which this occurred. According to Table 2, the PM_1.1_ at three of the industrial sampling sites at NJ, WH and NB and the background site UN had higher Phi values than the larger two fractions of PM_1.1–2.1_ and PM_2.1–9.0_ for the corresponding locations, suggesting that the fine atmospheric particles were the most polluted fractions, regardless of the industrial activity.

**Table 2:** PROMETHEE ranking of PM_2.1–9.0_, PM_1.1–2.1_, and PM_1.1_ at sampling sites KM, WH, NJ, NB and UN with Phi, Phi+ and Phi- values.

Rank	Samples	Locations	Phi	Phi+	Phi-
1	PM_1.1_	NJ	0.46	0.50	0.04
2	PM_1.1_	WH	0.38	0.42	0.04
3	PM_2.1–9.0_	KM	0.34	0.39	0.05
4	PM_1.1_	KM	0.24	0.30	0.06
5	PM_2.1–9.0_	WH	0.04	0.15	0.10
6	PM_1.1_	UN	-0.03	0.09	0.12
7	PM_1.1_	NB	-0.05	0.07	0.13
8	PM_1.1–2.1_	KM	-0.12	0.05	0.17
9	PM_2.1–9.0_	NB	-0.13	0.04	0.17
10	PM_2.1–9.0_	NJ	-0.14	0.04	0.18
11	PM_2.1–9.0_	UN	-0.15	0.03	0.18
12	PM_1.1–2.1_	WH	-0.15	0.03	0.18
13	PM_1.1–2.1_	NJ	-0.18	0.02	0.20
14	PM_1.1–2.1_	NB	-0.23	0.00	0.24
15	PM_1.1–2.1_	UN	-0.26	0.00	0.26

The GAIA analysis was used to reveal correlations between the trace elements and the associate sampling site. A decision axis (Pi) displayed in a GAIA plane was indicative of the most polluted sampling site. The length and quality numbers were used to evaluate the reliability of the result represented in a GAIA plane.

**Fig 2:**
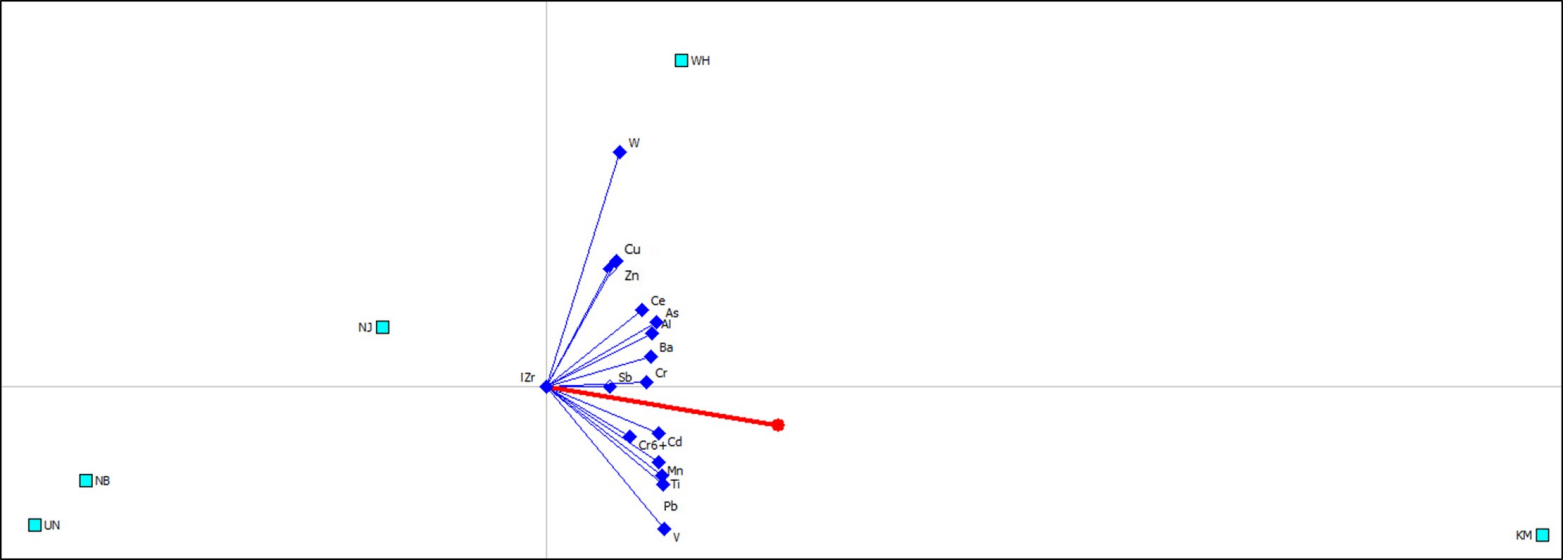
GAIA biplot for trace element contamination at atmospheric fraction of PM_2.1–9.0_ with quality of 94.5%. The decision axis (Pi) is displayed with a red line, and the ranking of Phil values obtained from PROMETHEE analysis at fives sampling sites is KM (0.7227) > WH (0.0805) > NJ (-0.1785) > NB (-0.2974) > UN (-0.3273).

Criteria vectors of the trace elements at the coarse fractions of PM_2.1–9.0_ were oriented in the same direction with the decision axis of Pi pointing towards the sampling site of KM (Fig 2). This suggests that KM had the most polluted PM_2.1–9.0_ particles than the other sampling sites, which was consistent with the PROMETHEE results which demonstrated that PM_2.1–9.0_ at KM had Phi value of 0.72 while the other samples had Phi values ranging from -0.33 to 0.08 (Fig 2). The industrial site NB and the background site UN were clearly grouped together and were in an obvious conflict with all criteria, indicating that both sites had the least polluted coarse particles.

**Fig 3:**
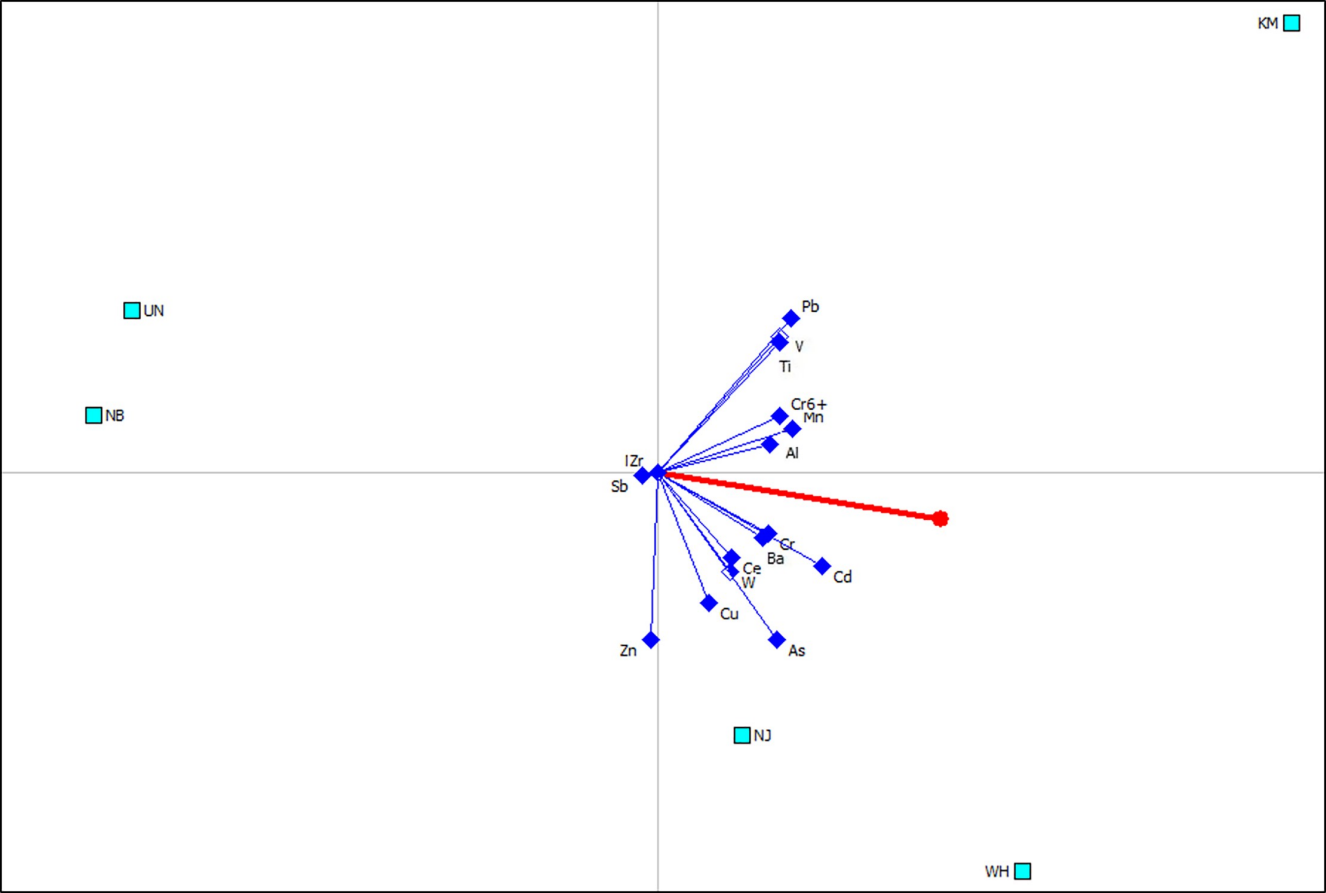
GAIA biplot for trace element contamination at atmospheric fraction of PM_1.1–2.1_ with quality of 82.5%. The decision axis (Pi) is displayed with a red line, and the ranking of Phil values obtained from PROMETHEE analysis at fives sampling sites is WH (0.4185) > KM (0.3059) > NJ (0.0932) > NB (-0.333) > UN (-0.4843).

Similarly, the sites NB and UN were also found to be located in the opposite direction to the Pi axis in Fig 3, suggesting that the fine PM_1.1–2.1_ particles at both sites were the least polluted amongst all the sampling sites. PM_1.1–2.1_ fractions for WH and KM had Phi values of 0.42 and 0.31, respectively, indicating pollution where the PM_1.1–2.1_ at WH were highly correlated with trace elements Cr, Cd, Ce, W, As and Cu, while at KM they were associated with Pb, V, Ti, Mn and Al (Fig 3).

**Fig 4:**
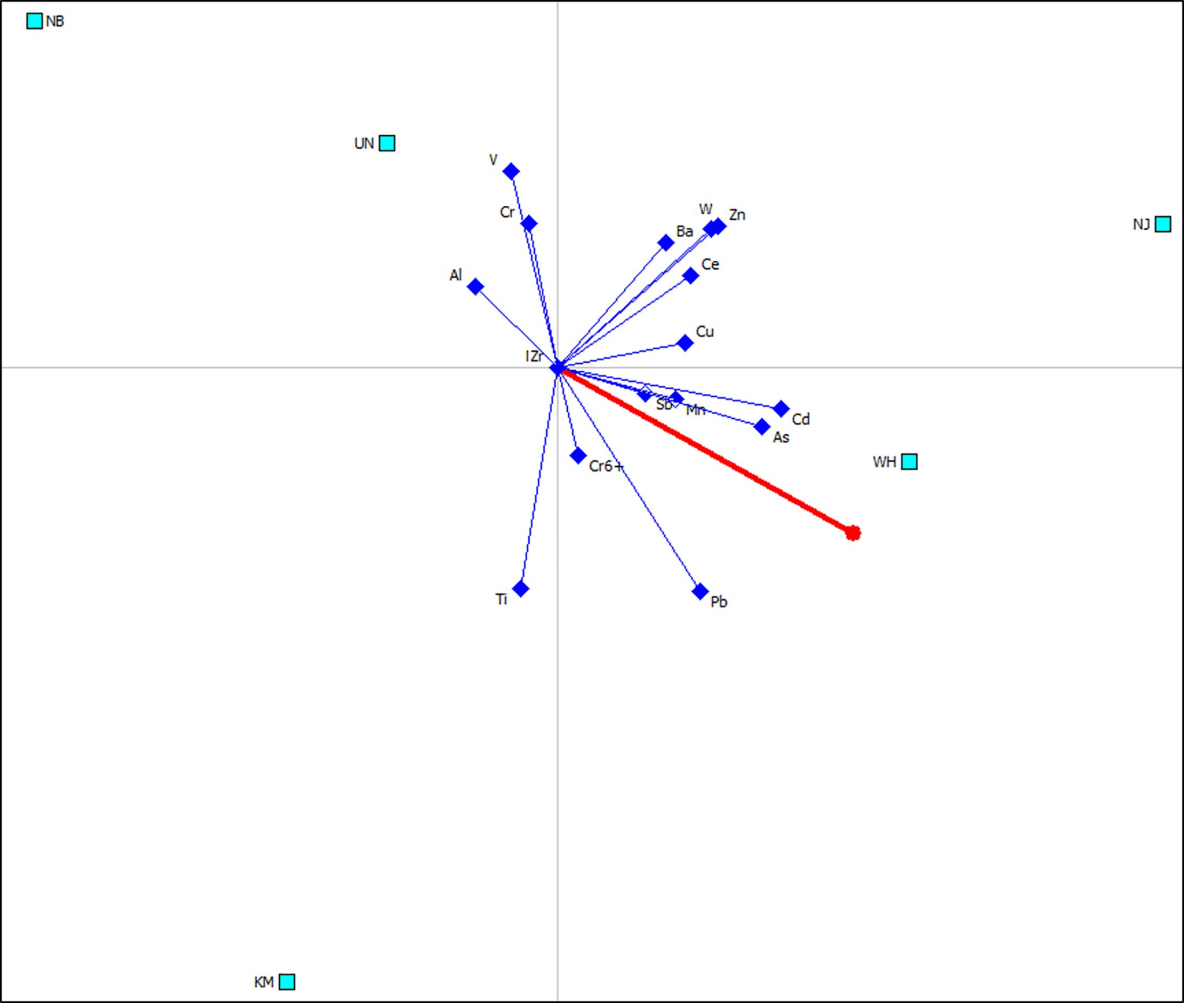
GAIA biplot for trace element contamination at atmospheric fraction of PM_1.1_ with quality of 73.2%. The decision axis (Pi) is displayed with a red line, and the ranking of Phil values obtained from PROMETHEE analysis at fives sampling sites is NJ (0.3045) > WH (0.2480) > KM (0.0421) > UN (-0.2911) > NB (-0.3035).

Compared to GAIA biplots of PM_2.1–9.0_ and PM_1.1–2.1_ (Figs 2 and 3), the GAIA results of PM_1.1_ had a more heterogeneous distribution of trace elements (Fig 4). The most polluted fraction of PM_1.1_ was found for the NJ site, which had the highest Phi value of 0.30 (Fig 4). The sampling site KM was independent from other sites and was associated with the trace element Ti (Fig 4). Trace elements As, Cd and Pb with long axes were close to the decision maker Pi, suggesting these elements played a significant role in the contamination levels of the fine particles.

### 3.2 Pb isotopic compositions

**Fig 5:**
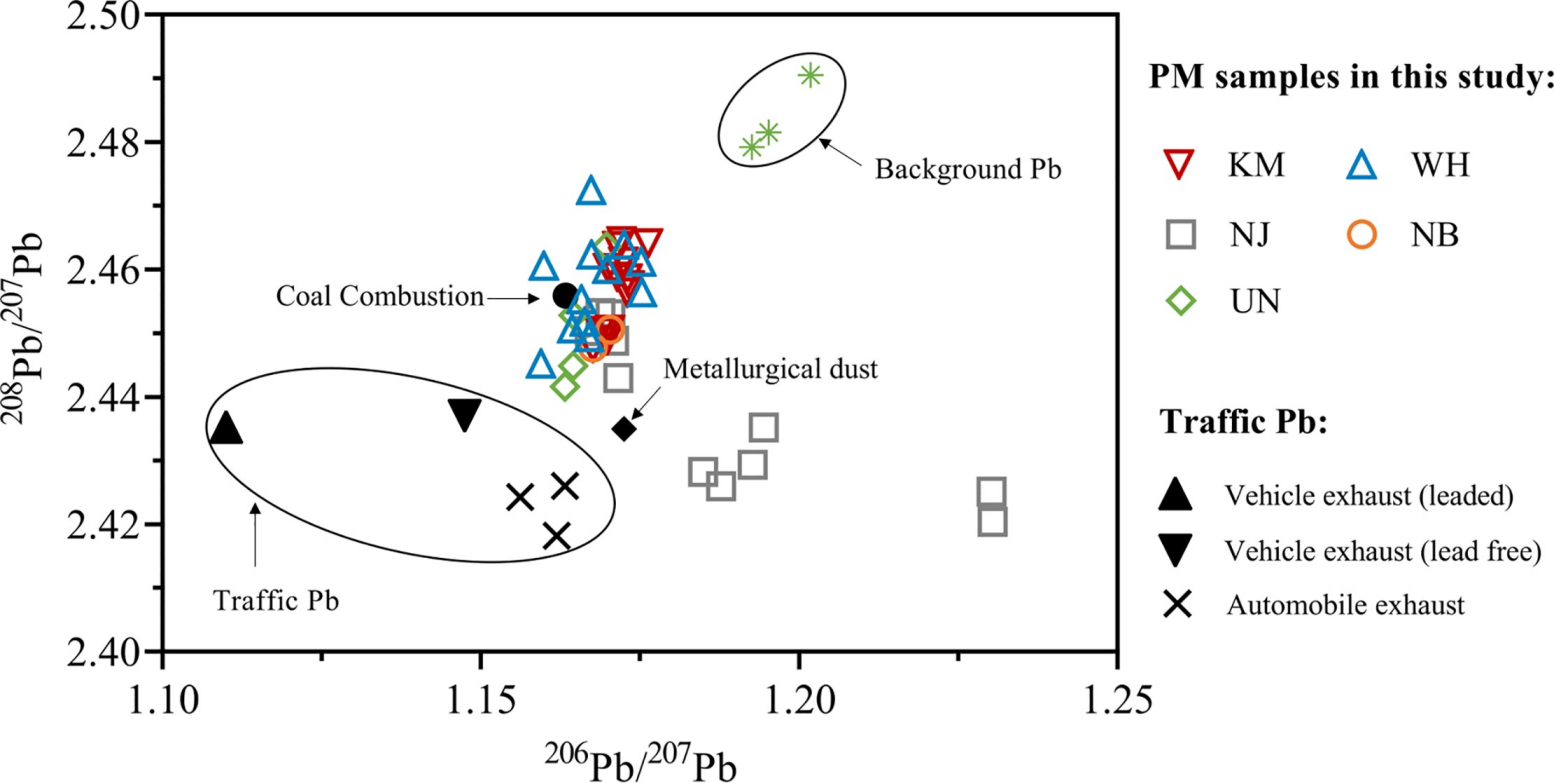
Lead isotopic compositions (^206^Pb/^207^Pb, ^208^Pb/^207^Pb) for PM samples collected at KM, WH, NJ, NB and UN. Lead isotopic compositions for background, coal combustion, metallurgical dust and traffic sources were obtained from Zhou et al. (2001) [46], and Tan [47].

The Pb compositions of ^206^Pb/^207^Pb and ^208^Pb/^207^Pb in the PM samples at all sampling sites are plotted in Fig 5. The PM data in this study was compared to other potential end-members, including uncontaminated background Pb [46], coal combustion [47], metallurgical dust [47], as well as traffic emissions associated with leaded petrol [47], unleaded petrol [47] and other automobile exhaust [46].

The Pb isotopic compositions of atmospheric particles collected near the iron and steelmaking areas of KM, WH, NJ and NB were distinct from the background Pb and traffic Pb, but very close to the Pb signatures of coal combustion and metallurgical dust (Fig 5).

**Fig 6:**
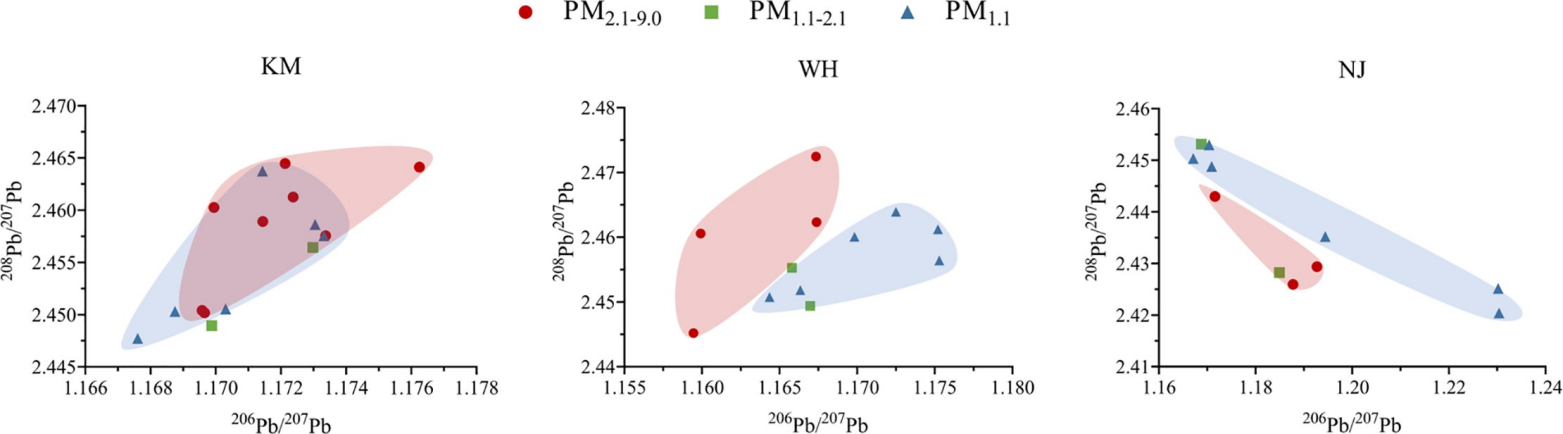
Lead isotopic compositions (^206^Pb/^207^Pb, ^208^Pb/^207^Pb) for different atmospheric fractions of PM_2.1–9.0_, PM_1.1–2.1_ and PM_1.1_ at sampling sites of KM, WH and NJ.

In order to determine Pb sources across different particle fractions, the Pb isotopic compositions of ^206^Pb/^207^Pb and ^208^Pb/^207^Pb at PM_2.1–9.0_, PM_1.1–2.1_ and PM_1.1_ collected from KM, WH and NJ were plotted in Fig 6. The Pb isotopic compositions of PM_2.1–9.0_, PM_1.1–2.1_ and PM_1.1_ at KM site were overlapped, suggesting that the Pb contamination across three fractions at KM were dominated by the same source, such as the local steelmaking activities. However, the Pb isotopic compositions at PM_2.1–9.0_ and PM_1.1_ fractions showed distinct differences at the sampling sites of WH and NJ, indicating that the coarse and fine particles have different dominant Pb contamination sources.

### 3.3 Health risks

#### 3.3.1 Inhalation risks

**Fig 7:**
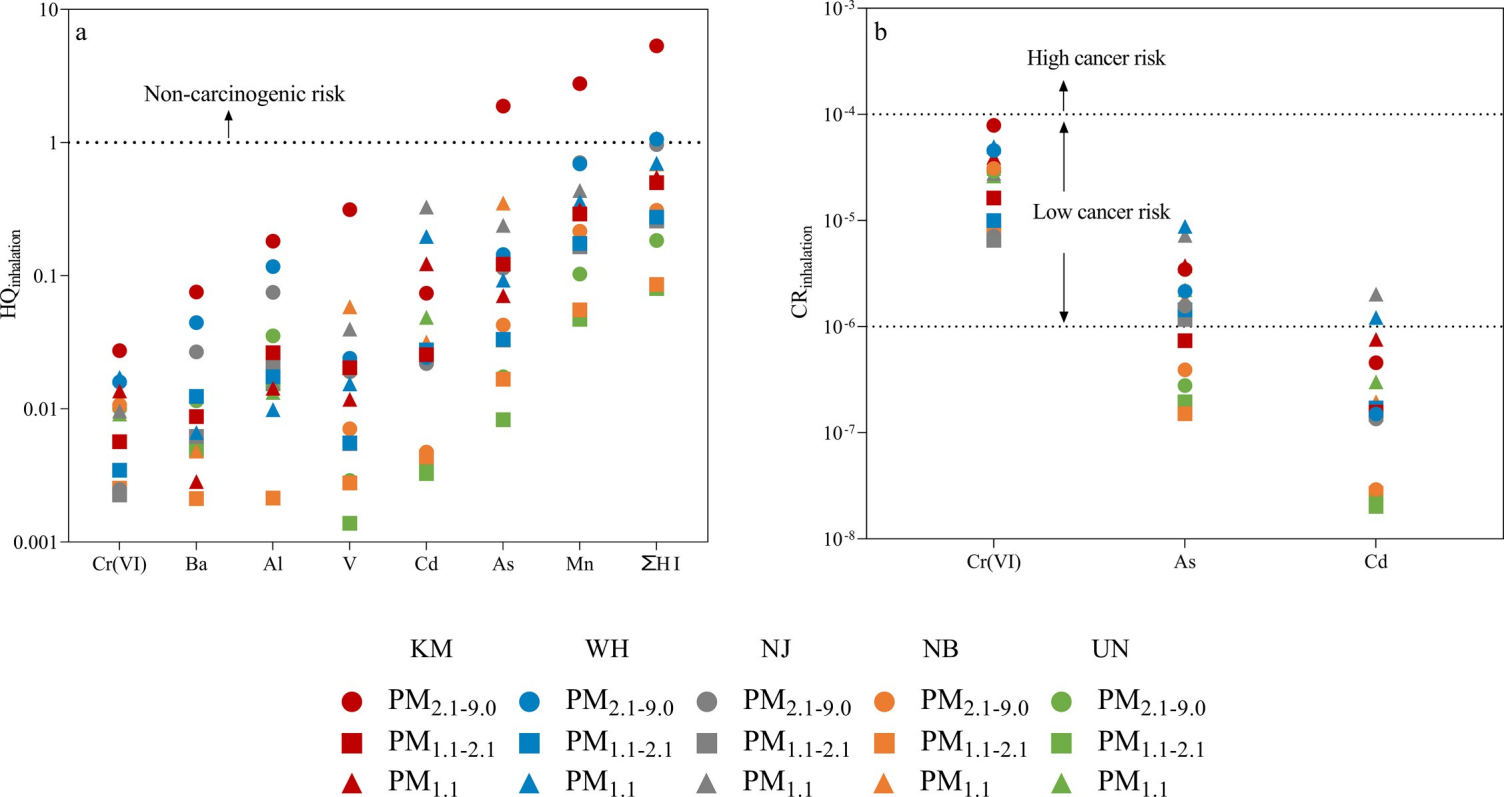
Non-carcinogenic risk and carcinogenic risk via inhalation exposure at fractions of PM_2.1–9.0_, PM_1.1–2.1_ and PM_1.1_ across five sampling sites. The absence of symbols for HQ and CR values was due to the corresponding trace elements with concentrations < LOR.

According to Eqs 3 and 4, the reference values of RfCi were used to calculate non-carcinogenic risks (HQ) for each trace element. The RfCi for trace elements Al, As (inorganic), Ba, Cd, Cr(VI), Mn and V were 5.0, 0.015, 0.5, 0.01, 0.1, 0.05 and 0.1 μg/m^3^, respectively [44], and their corresponding non-carcinogenic risks (HQ and ∑HQ) were calculated for three atmospheric fractions (PM_2.1–9.0_, PM_1.1–2.1_ and PM_1.1_) at five sampling sites (Fig 7A).

The highest ∑HQ value of 5.32 was found for the fraction of PM_2.1–9.0_ collected from KM, with As and Mn as the dominant contributing trace elements which had HQ values of 1.88 and 2.77, respectively (Fig 7A). These values were much higher than the safe level of HQ = 1, suggesting that concentrations of As and Mn coupled with other trace elements at PM_2.1–9.0_ at KM posed a significant non-carcinogenic risk.

The coarse fraction of PM_2.1–9.0_ at WH (∑HQ = 1.06) and fine fraction of PM_1.1_ at NJ (∑HQ = 1.08) also had ∑HQ values beyond the limit of one, but there was no individual trace element with HQ > 1. This suggests that trace element concentrations of PM_2.1–9.0_ at WH and PM_1.1_ at NJ can result in the accumulative non-carcinogenic health risk for residents via inhalation exposure.

Cancer risks via inhalation exposure were calculated and displayed in Fig 7B. The IUR values for trace elements As (inorganic), Cd and Cr(VI) are 4.3E-03, 1.8E-03 and 8.4E-02 (μg/m^3^)^-1^, respectively [44], and hence these three elements were used to assess the potential carcinogenic risks. In order to determine a relative conservative health risk caused by As, the total concentrations of As measured in this study were assumed to be in the inorganic form [36], which is considered to be associated with cardiovascular disease and diabetes [48].

The CR value between 10^−6^ and 10^−4^ is considered to pose a potential cancer risk and CR values with > 10^−4^ are considered to highly likely cause cancer. In this study, apart from the Cr(VI) of PM_1.1–2.1_ fraction at NB site with a concentration < LOR and not used for cancer risk assessment, Cr(VI) concentrations of the other samples presented high CR values exceeding the limit of 10^−6^ (Fig 7B). This suggests that airborne Cr(VI) near the iron and steelmaking plants investigated in this work pose a likely cancer risk to humans.

In addition to Cr(VI), As was also found to have CR values greater than 10^−6^ at different PM fractions, especially in the fine particle of PM_1.1_. The PM_1.1_ samples at all the sampling sites were found to have carcinogenic risks as a result of As contamination. The health risks caused by Cd contamination were also found at PM_1.1_ particles (Fig 7B), indicating that the fine particles posed more adverse inhalation risks than coarse particles.

#### 3.3.2 Ingestion risks

**Fig 8:**
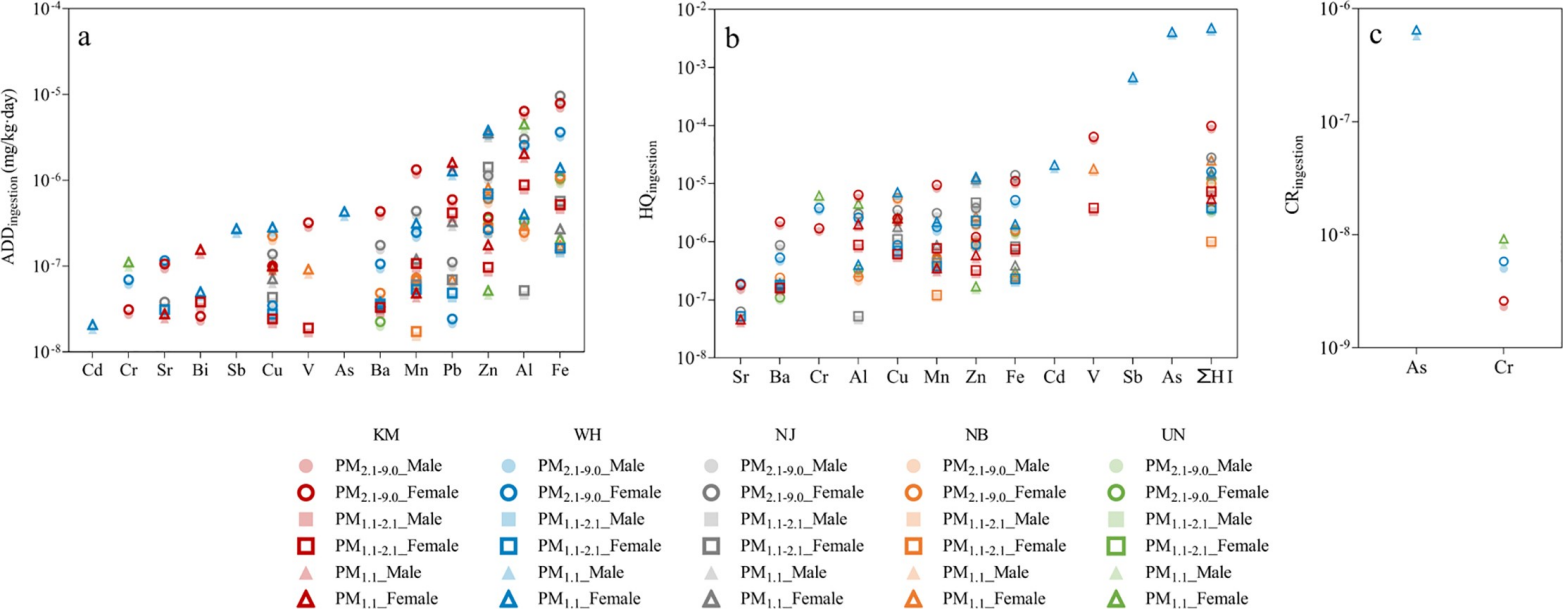
Data for average daily dose (ADD), hazard quotient (HQ) and cancer risk (CR) via ingestion exposure for PM_2.1–9.0_, PM_1.1–2.1_ and PM_1.1_ across five sampling sites. The absence of symbols for ADD, HQ and CR values was due to the corresponding trace elements with concentrations < LOR. The health risks caused by Cr contamination were calculated by 1/6^th^ of the total bioavailable Cr concentrations, while the total bioavailable As concentrations were assumed as an inorganic form [36].

Trace element concentrations extracted from the bioavailability analysis were used to assess the non-carcinogenic and carcinogenic health risks via ingestion exposure. The parameter of body weight (BW) used to calculate the health risks were different for males and females, hence the average daily dose (ADD), hazard quotient (HQ) and cancer risk (CR) values were displayed according to genders (Fig 8A–8C). The results showed that Mn, Pb, Zn, Al and Fe were the dominant trace elements for ADD ingestion values, and that females tend to have a higher exposure dose via ingestion than males (Fig 8A). As a result, females experienced slightly higher non-carcinogenic risks than males (Fig 8B).

The HQ results suggested that the fine particles PM_1.1_ had the highest HQ values, although their HQ values and the sum of HQ values did not exceed the safety limit of HQ = 1. A similar trend was also found for carcinogenic risk assessment. The PM_1.1_ particles had higher CR values than PM_1.1–2.1_ and PM_2.1–9.0_ particles for both genders, although their values were within the acceptable limit of 10^−6^ (Fig 8C).

## Discussion

The iron and steelmaking activities are considered as a significant contributor to the state of local air quality in China [13]. This study showed that trace element concentrations at PM particles collected from intensive steelmaking areas in China were elevated with higher risks to human health than samples at the background site located away from industrial activities (S1 Table, Figs 7 and 8). For example, the trace elements As and Mn in PM_2.1–9.0_ fractions at KM site were 12 and 27 times higher than the corresponding concentrations of the background UN sample. As a result, the elevated As and Mn contents at PM_2.1–9.0_ of KM were found to exceed the safety limits, presenting non-carcinogenic risks via inhalation exposure (Fig 7A) [49].

Compared to the air quality at steelmaking areas of KM, WH and NJ, PM particles collected near the steel mill at NB had low trace element concentrations, which were similar to the levels at the background area of UN (S1 Table). The PROMETHEE and GAIA results clearly showed that the sampling sites of NB and the background UN can be grouped into a same cluster for the three fractions of PM_2.1–9.0_ (Fig 2), PM_1.1–2.1_ (Fig 3) and PM_1.1_ (Fig 4) because the sampling site at NB was located at the upwind of the local steel mill (Table 1), and was approximately 40 km away from the UN site.

The trace element analysis also showed that the small PM particles tend to be more contaminated than the coarse fractions [50]. This was also found in the PROMETHEE results which showed that the fine particles at PM_1.1_ size had higher Phi values than the other two fractions (Table 2), suggesting higher enrichment by trace elements. As a result, fine particles of PM_1.1_ presented a higher health risk than PM_1.1–2.1_ and PM_2.1–9.0_ [33]. For example, concentrations of As, Cd and Cr(VI) at PM_1.1_ fractions across the steelmaking areas had CR values up to 4.96E-05, indicating that approximately five people out of a population of 100,000 have possibility to develop cancer during the lifetime of 70 years.

The PM particles incorporated with trace elements are generated during various stages of the steelmaking process [51]. The BOF and BF as key facilities for steelmaking are considered as a large contributor to PM emissions [52]. The steel mill at WH investigated in this study has the maximum number of furnaces (8 BFs and 10 BOFs) with the largest capacity for steel production of up to 20 million tonnes per year among the sampled plants (Table 1). Consequently, the sampling site of WH presented acute angles with the decision axis Pi in GAIA biplots, suggesting PM particles at WH were highly contaminated by trace elements across the three fractions, especially PM_1.1–2.1_ and PM_1.1_ (Figs 3 and 4). GAIA results further showed that PM emission at WH were significantly associated with As and Cd at fine particles as both elements presented long criterial axes pointing towards WH (Figs 3 and 4). Previous studies have evidenced that As and Cd emissions at fine PM particles were related to metal smelting and fuel combustion under high temperature processing [39]. Previous studies also showed that chemical compositions of airborne particle emissions varied with steelmaking facilities [53]. For example, compared to BOF dust, which was estimated to contain 2–8% Zn [54], the EAF dust contains Zn up to 19.4% [55]. This high correlation between Zn contamination and EAF facility was also observed in this study. The only EAF equipment in this study was located at the NJ steel mill (Table 1), and the vector of Zn concentrations presented a long criterial axis highly related to its local PM_1.1–2.1_ and PM_1.1_ fractions (Figs 3 and 4).

Although iron and steelmaking activities were a dominant source to PM emissions, traffic emission also played a significant role in local air quality [56]. Traffic emissions related to brake wear, road abrasion and dust resuspension as a result of the mechanical process are considered a significant contributor to PM coarse fractions [57], which is also relevant to this study. At the KM site, the sampling location was next to a main road with high traffic volume, while sampling locations at WH, NJ and NB sites were far away from other contamination sources. As a result, the coarse fractions of PM_2.1–9.0_ at KM had higher trace element concentrations with higher Phi value (Phi = 0.34) than PM_2.1–9.0_ particles at other sampling sites (Phi = -0.15–0.04) (Table 2). The GAIA results also showed that the sampling site of KM was consistent with the decision axis Pi and far away from other sites at PM_2.1–9.0_ fraction (Fig 2). This suggests that the coarse fraction at KM was most contaminated among the sampling sites as a result of local contamination sources of both iron and steelmaking activities and heavy traffic.

In addition to the local contamination sources, PM particles can also be transferred from the remote pollution sources (S1 Fig), particularly the fine PM_1.1_ particles which can travel up to tens of kilometres [58]. The remote contributor to PM_1.1_ contamination was also observed in this study. Compared to coarse fraction of PM_2.1–9.0_ and intermodal PM_1.1–2.1_, the small particles of PM_1.1_ had a more heterogenous distribution of trace elements in the GAIA plot (Fig 4 vs Figs 2 and 3). This indicated relatively low interrelationships between trace elements at PM_1.1_ fractions due to various remote sources in addition to local iron and steelmaking contamination.

The main driving factor bringing remote pollution into the local environment is hypothesised to be wind (S1 Fig). Compared to other sampling sites with clear weather during the sampling period, the NJ site experienced strong winds with the maximum speed of 6.8 m/s and frequencies ranging from 2.8 to 5.5 m/s (Fig 1). As a result, the particles in this site had larger ^206^Pb/^207^Pb and ^208^Pb/^207^Pb range at PM_1.1_ than in PM_2.1–9.0_, suggesting that Pb contamination in the fine PM_1.1_ particles at NJ was not only dominated by local iron and steelmaking activities but contributed by remote contamination through the forces of strong winds.

## Conclusion

This study collected PM particles across different sizes near iron and steelmaking areas in China and characterized the PM fractions with multiple lines of chemical and statistical analyses. Due to the complexity of performing atmospheric sampling in industrial areas in China, the objectives of the study were limited to assessment of the application of the selected analytical and modelling research methods to study differences of atmospheric particles in industrial areas with variable age and pollution controls over a one week period of sampling time. The study characterized PM contamination in typical Chinese steel cities with spatial significance. Four sampling cities were located at the Yangtze River Economic Zone (YREZ) which accounts for 20% of national Gross Domestic Product and is responsible for 1/3 of China’s imports and exports. The cities selected in this study across southwest (Kunming), central (Wuhan) and east regions (Nanjing and Ningbo) covered developed and less developed areas in China. The sampling in this study was conducted during late April to early July when there was the shifting period from spring to summer in China. During this period, the weather was clear with moderate temperatures and humidity which was suitable for atmospheric particle sampling. The air pollution sources were relatively limited compared to wintertime which is typically dominated by coal combustion for heating purpose.

The results in this study showed that the PM emissions from iron and steelmaking process were associated with the age of the facilities, facility types and the capabilities of annual production of iron and steel. The trace element results showed that the small PM_1.1_ particles were more contaminated than the intermodal and coarse fractions. As a result, PM_1.1_ particles posed a negative impact on human health, specifically trace elements of As, Cr(VI) and Cd which presented carcinogenic risks via inhalation exposure. The Pb isotopic compositions showed that the local iron and steelmaking activities were the major source of PM contamination, followed by traffic and unidentified remote sources due to atmospheric movement. Results presented in this study implicate that sampling over a longer period is required to monitor air quality near industrial areas in order to confirm the impact of meteorological conditions on the transportation of atmospheric particles from the industrial sources.

## Supporting information

S1 Fig HYSPLIT backward trajectories at sites of Kunming (KM), Wuhan (WH), Nanjing (NJ), Ningbo (NB) and Ningbo Nottingham University (UN) during sampling periods in this study. (DOCX)

S1 Table Modelling parameters for PROMETHEE and GAIA analyse. (DOCX)

S2 Table Bioavailability analysis for trace elements. (DOCX)

## References

[1] Maytaal, A. 2018. Worldsteel raises forecast for 2018 global steel demand growth to 1.8 percent. [Accessed: 2019 July 4]; Available from: https://www.reuters.com/article/us-global-steel-demand/worldsteel-raises-forecast-for-2018-global-steel-demand-growth-to-1-8-percent-idUSKBN1HO1DY.

[2] Worldsteel Association, Fact Sheet—Steel and raw materials. 2019. p. 3.

[3] Ogundele L.T., Owoade O.K., Hopke P.K., and Olise F.S., Heavy metals in industrially emitted particulate matter in Ile-Ife, Nigeria. Environ. Res., 2017 156: p. 320–325. 10.1016/j.envres.2017.03.051 28390299

[4] Liu W., Xu Y., Zhao Y., Liu Q., Yu S., et al Occurrence, source, and risk assessment of atmospheric parent polycyclic aromatic hydrocarbons in the coastal cities of the Bohai and Yellow Seas, China. Environ. Pollut., 2019 10.1016/j.envpol.2019.113046 31454587

[5] Liu Q., Lu Z., Xiong Y., Huang F., Zhou J., and Schauer J.J. Oxidative potential of ambient PM2.5 in Wuhan and its comparisons with eight areas of China. Sci. Total Environ., 2020 10.1016/j.scitotenv.2019.134844 31704396

[6] WHO, Health Risks of Heavy Metals From Long-Range Transboundary Air Pollution. 2007: Germany. p. 144.

[7] Hemon W.C.L., Air Pollution Problemsof theSteel Industry. J. Air Pollut. Control Assoc., 1960 10(3): p. 208–253.

[8] Mohamed J. and Sasi B., Air pollution caused by iron and steel plants. IJMMME, 2013 1(3): p. 219–222.

[9] Heiden P.I.D. and Taube M., China's iron and steel industry at the global markets interface: Structural developments and industrial policy interventions. CJAS, 2011 29(2): p. 110–142.

[10] Worldsteel Association. 2019. Global crude steel output increases by 4.6% in 2018. [Accessed: 2019 July 4]; Available from: https://www.worldsteel.org/media-centre/press-releases/2019/Global-crude-steel-output-increases-by-4.6—in-2018.html.

[11] National Bureau of Statistics. 2018. Dust (soot) emission (tons) 烟(粉)尘排放量(吨). [Accessed: 2019 July 4]; Available from: http://www.stats.gov.cn/.

[12] Li M., Liu H., Geng G., Hong C., Liu F., Song Y., et al, Anthropogenic emission inventories in China: A review. Natl. Sci. Rev., 2017 4(6): p. 834–866.

[13] Deng, Q. 2019. Air quality at the top 20 steelmaking cities below the national standard 我国钢铁产能排名前20位的城市无一空气质量达标. [Accessed: 2019 July 4]; Available from: https://xw.qq.com/cmsid/20190506A0K0T100.

[14] Brunekreef B. and Holgate S.T., Air pollution and health. Lancet, 2002 360(9341): p. 1233–1242. 10.1016/S0140-6736(02)11274-8 12401268

[15] Mateos A.C., Amarillo A.C., Carreras H.A., and Gonzalez C.M., Land use and air quality in urban environments: Human health risk assessment due to inhalation of airborne particles. Environ. Res., 2018 161: p. 370–380. 10.1016/j.envres.2017.11.035 29197278

[16] Allen A.G., Nemitz E., Shi J.P., Harrison R.M., and Greenwood J.C., Size distributions of trace metals in atmospheric aerosols in the United Kingdom. Atmos. Environ., 2001 35(27): p. 4581–4591.

[17] Geller M.D., Sardar S.B., Phuleria H., Fine P.M., and Sioutas C., Measurements of particle number and mass concentrations and size distributions in a tunnel environment. Environ. Sci. Technol., 2005 39(22): p. 8653–8663. 10.1021/es050360s 16323759

[18] Sanchez-Soberon F., Rovira J., Sierra J., Mari M., Domingo J.L., and Schuhmacher M., Seasonal characterization and dosimetry-assisted risk assessment of indoor particulate matter (PM_10-2.5_, PM_2.5–0.25_, and PM_0.25_) collected in different schools. Environ. Res., 2019 175: p. 287–296. 10.1016/j.envres.2019.05.035 31146100

[19] Loxham M., Cooper M.J., Gerlofs-Nijland M.E., Cassee F.R., Davies D.E., Palmer M.R., et al, Physicochemical characterization of airborne particulate matter at a mainline underground railway station. Environ. Sci. Technol., 2013 47(8): p. 3614–22. 10.1021/es304481m 23477491 PMC3687366

[20] Hieu N.T. and Lee B.-K., Characteristics of particulate matter and metals in the ambient air from a residential area in the largest industrial city in Korea. Atmos. Res., 2010 98(2–4): p. 526–537.

[21] Lin C.-C., Chen S.-J., Huang K.-L., Hwang W.-I., Chang-Chien G.-P., and Lin W.-Y., Characteristics of metals in nano/ultrafine/fine/coarse particles collected beside a heavily trafficked road. Environ. Sci. Technol., 2005 39(21): p. 8113–8122. 10.1021/es048182a 16294844

[22] Nikolic D., Jovanovic I., Mihajlovic I., and Zivkovic Z., Multi-criteria ranking of copper concentrates according to their quality—an element of environmental management in the vicinity of copper—smelting complex in Bor, Serbia. J. Environ. Manage., 2009 91(2): p. 509–15. 10.1016/j.jenvman.2009.09.019 19833429

[23] Ilić I., Bogdanović D., Živković D., Milošević N., and Todorović B., Optimization of heavy metals total emission, case study: Bor (Serbia). Atmos. Res., 2011 101(1–2): p. 450–459.

[24] Herngren L., Goonetilleke A., and Ayoko G.A., Analysis of heavy metals in road-deposited sediments. Anal. Chim. Acta, 2006 571(2): p. 270–8. 10.1016/j.aca.2006.04.064 17723448

[25] Gunawardena J., Egodawatta P., Ayoko G.A., and Goonetilleke A., Atmospheric deposition as a source of heavy metals in urban stormwater. Atmos. Environ., 2013 68: p. 235–242.

[26] Mahbub P., Ayoko G.A., Goonetilleke A., Egodawatta P., and Kokot S., Impacts of traffic and rainfall characteristics on heavy metals build-up and wash-off from urban roads. Environ. Sci. Technol., 2010 44(23): p. 8904–10. 10.1021/es1012565 20964357

[27] Gunawardana C., Egodawatta P., and Goonetilleke A., Adsorption and mobility of metals in build-up on road surfaces. Chemosphere, 2015 119: p. 1391–1398. 10.1016/j.chemosphere.2014.02.048 24630452

[28] Kot A. and Namiesńik J., The role of speciation in analytical chemistry. Trends Anal. Chem., 2000 19(2–3): p. 69–79.

[29] Jobby R., Jha P., Yadav A.K., and Desai N., Biosorption and biotransformation of hexavalent chromium [Cr(VI)]: A comprehensive review. Chemosphere, 2018 207: p. 255–266. 10.1016/j.chemosphere.2018.05.050 29803157

[30] Ma H.W., Hung M.L., and Chen P.C., A systemic health risk assessment for the chromium cycle in Taiwan. Environ. Int., 2007 33(2): p. 206–18. 10.1016/j.envint.2006.09.011 17074391

[31] U.S. EPA. 1986. Risk Assessment for Carcinogenic Effects. [Accessed: 2019 July 4]; Available from: https://www.epa.gov/fera/risk-assessment-carcinogenic-effects.

[32] Rovira J., Vilavert L., Nadal M., Schuhmacher M., and Domingo J.L., Temporal trends in the levels of metals, PCDD/Fs and PCBs in the vicinity of a municipal solid waste incinerator. Preliminary assessment of human health risks. Waste Manag., 2015 43: p. 168–75. 10.1016/j.wasman.2015.05.039 26130170

[33] Sánchez-Soberón F., Rovira J., Mari M., Sierra J., Nadal M., Domingo J.L., et al, Main components and human health risks assessment of PM_10_, PM_2.5_, and PM_1_ in two areas influenced by cement plants. Atmos. Environ., 2015 120: p. 109–116.

[34] Rovira J., Nadal M., Schuhmacher M., and Domingo J.L., Alternative fuel implementation in a cement plant: Human health risks and economical valuation. Arch. Environ. Contam. Toxicol., 2016 71(4): p. 473–484. 10.1007/s00244-016-0308-2 27558466

[35] Galindo N., Yubero E., Nicolas J.F., Varea M., and Crespo J., Characterization of metals in PM_1_ and PM_10_ and health risk evaluation at an urban site in the western Mediterranean. Chemosphere, 2018 201: p. 243–250. 10.1016/j.chemosphere.2018.02.162 29524825

[36] Rovira J., Nadal M., Schuhmacher M., and Domingo J.L., Concentrations of trace elements and PCDD/Fs around a municipal solid waste incinerator in Girona (Catalonia, Spain). Human health risks for the population living in the neighborhood. Sci. Total Environ., 2018 630: p. 34–45. 10.1016/j.scitotenv.2018.02.175 29471189

[37] Pena-Fernandez A., Gonzalez-Munoz M.J., and Lobo-Bedmar M.C., Establishing the importance of human health risk assessment for metals and metalloids in urban environments. Environ. Int., 2014 72: p. 176–85. 10.1016/j.envint.2014.04.007 24791693

[38] Li S.W., Li H.B., Luo J., Li H.M., Qian X., Liu M.M., et al, Influence of pollution control on lead inhalation bioaccessibility in PM_2.5_: A case study of 2014 Youth Olympic Games in Nanjing. Environ. Int., 2016 94: p. 69–75. 10.1016/j.envint.2016.05.010 27209002

[39] Yang X., Zhou X., Kan T., Strezov V., Nelson P., Evans T., et al Characterization of size resolved atmospheric particles in the vicinity of iron and steelmaking industries in China. Sci. Total Environ., 2019 10.1016/j.scitotenv.2019.07.340 31756840

[40] Espinasse B., Picolet G., and Chouraqui E., Negotiation support systems: A multi-criteria and multi-agent approach. Eur. J. Oper. Res., 1997 103(2): p. 389–409.

[41] Liu L., Liu A., Li Y., Zhang L., Zhang G., and Guan Y., Polycyclic aromatic hydrocarbons associated with road deposited solid and their ecological risk: Implications for road stormwater reuse. Sci. Total Environ., 2016 563–564: p. 190–8. 10.1016/j.scitotenv.2016.04.114 27135582

[42] U.S. EPA, Supplemental Guidance For Developing Soil Screening Levels For Superfund Sites. 2002: Washington, D.C. p. 187.

[43] Zhao, X., L. Chen, B. Wang, N. Huang, S. Cao, T. Dong, et al., 中国人群暴露参数手册(成人卷)Highlights of the chinese exposure factors handbook (adults). 2014, Ministry of Ecology and Environment of the People's Republic of China: Beijing. p. 71.

[44] U.S. EPA. 2019. Regional Screening Levels (RSLs)—Generic Tables. [Accessed: 2019 July 3]; Available from: https://www.epa.gov/risk/regional-screening-levels-rsls-generic-tables.

[45] Megido L., Suarez-Pena B., Negral L., Castrillon L., and Fernandez-Nava Y., Suburban air quality: Human health hazard assessment of potentially toxic elements in PM10. Chemosphere, 2017 177: p. 284–291. 10.1016/j.chemosphere.2017.03.009 28314233

[46] Zhu B.-Q., Chen Y.-W., and Peng J.-H., Lead isotope geochemistry of the urban environment in the Pearl River Delta. Appl. Geochem., 2001 16(4): p. 409–417.

[47] Tan M.G., Zhang G.L., Li X.L., Zhang Y.X., Yue W.S., Chen J.M., et al, Comprehensive study of lead pollution in Shanghai by multiple techniques. Anal. Chem., 2006 78(23): p. 8044–8050. 10.1021/ac061365q 17134138

[48] WHO. 2018. Arsenic. [Accessed: 2019 July 4]; Available from: https://www.who.int/news-room/fact-sheets/detail/arsenic.

[49] Hernandez-Pellon A., Nischkauer W., Limbeck A., and Fernandez-Olmo I., Metal(loid) bioaccessibility and inhalation risk assessment: A comparison between an urban and an industrial area. Environ. Res., 2018 165: p. 140–149. 10.1016/j.envres.2018.04.014 29704775

[50] Fang W., Yang Y., and Xu Z., PM_10_ and PM_2.5_ and health risk assessment for heavy metals in a typical factory for cathode ray tube television recycling. Environ. Sci. Technol., 2013 47(21): p. 12469–12476. 10.1021/es4026613 24083671

[51] Tsai J., Lin K., Chen C., Ding J., Choa C., and Chiang H., Chemical constituents in particulate emissions from an integrated iron and steel facility. J. Hazard. Mater., 2007 147(1–2): p. 111–119. 10.1016/j.jhazmat.2006.12.054 17276592

[52] Taiwo A.M., Beddows D.C.S., Calzolai G., Harrison R.M., Lucarelli F., Nava S., et al, Receptor modelling of airborne particulate matter in the vicinity of a major steelworks site. Sci. Total Environ., 2014 490: p. 488–500. 10.1016/j.scitotenv.2014.04.118 24875261

[53] Dall'Osto M., Booth M.J., Smith W., Fisher R., and Harrison R.M., A study of the size distributions and the chemical characterization of airborne particles in the vicinity of a large integrated steelworks. Aerosol Sci. Tech., 2008 42(12): p. 981–991.

[54] Nyirenda R.L., The processing of steelmaking flue-dust: A review. Miner. Eng., 1991 4(7–11): p. 1003–1025.

[55] Jha M.K., Review of hydrometallurgical recovery of zinc from industrial wastes. Resour. Conserv. Recy., 2001 33: p. 1–22.

[56] Rovira J., Sierra J., Nadal M., Schuhmacher M., and Domingo J.L., Main components of PM_10_ in an area influenced by a cement plant in Catalonia, Spain: Seasonal and daily variations. Environ. Res., 2018 165: p. 201–209. 10.1016/j.envres.2018.04.010 29727820

[57] Thorpe A. and Harrison R.M., Sources and properties of non-exhaust particulate matter from road traffic: a review. Sci. Total Environ., 2008 400: p. 270–282. 10.1016/j.scitotenv.2008.06.007 18635248

[58] WHO, Health Risks of Particulate Matter From Long-Range Transboundary Air Pollution. 2006: Denmark. p. 113.

