# Supplementary material for: Contamination identification, source apportionment and health risk assessment of trace elements at different fractions of atmospheric particles at iron and steelmaking areas in China
Source: PLoS One. 2020 Apr 2;15(4):e0230983. doi: 10.1371/journal.pone.0230983 (PMC7117772; doi:10.1371/journal.pone.0230983)
Supplement: S1 Fig — (DOCX) [file pone.0230983.s001.docx]

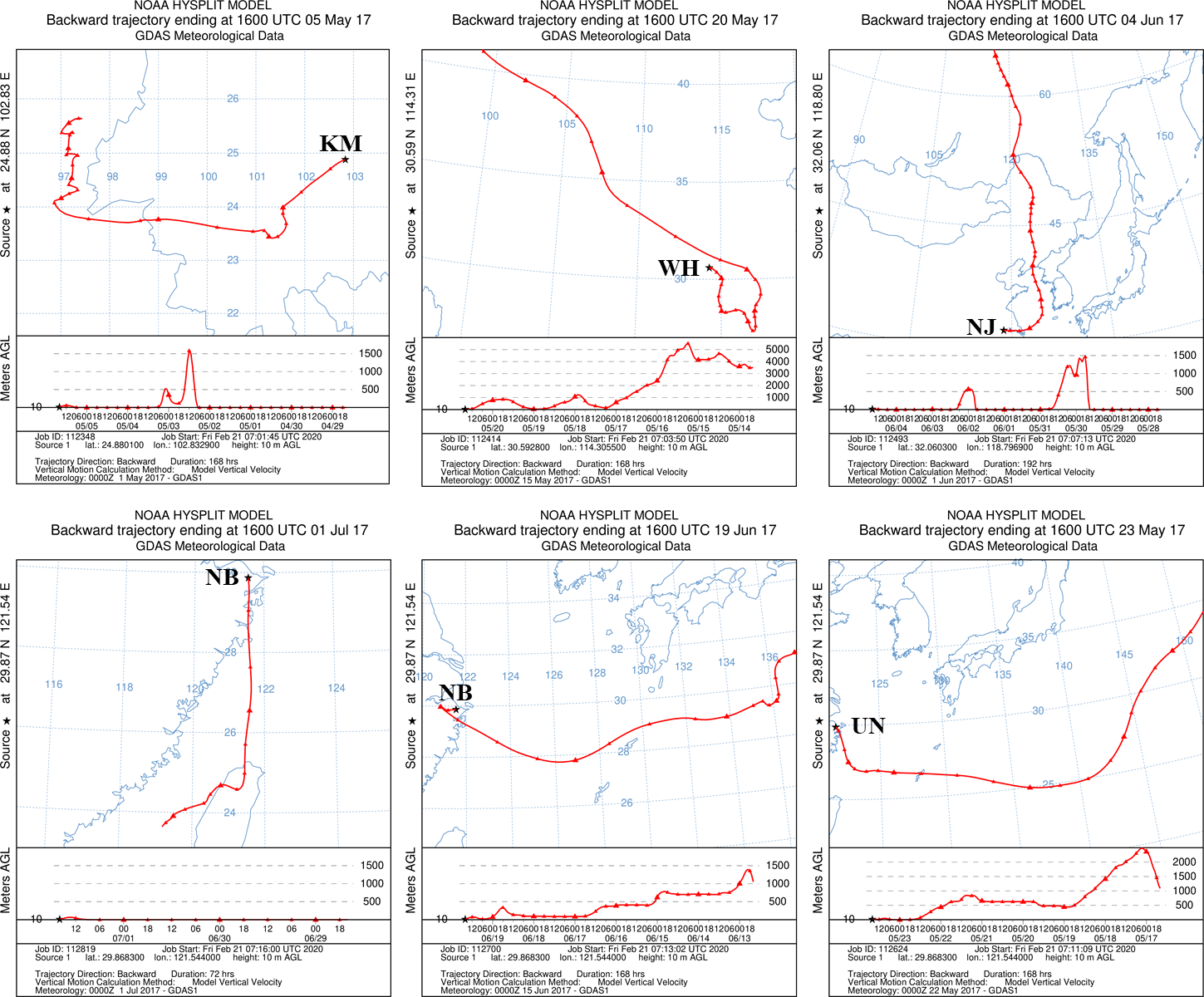


**S1 Fig**. HYSPLIT backward trajectories at sites of Kunming (KM), Wuhan (WH), Nanjing (NJ), Ningbo (NB) and Ningbo Nottingham University (UN) during sampling periods in this study. Air filter samples at NB site were collected during two periods due to wet weather disruption [1].
