# Supplementary material for: Contamination identification, source apportionment and health risk assessment of trace elements at different fractions of atmospheric particles at iron and steelmaking areas in China
Source: PLoS One. 2020 Apr 2;15(4):e0230983. doi: 10.1371/journal.pone.0230983 (PMC7117772; doi:10.1371/journal.pone.0230983)
Supplement: S1 Table — (DOCX) [file pone.0230983.s002.docx]

**S1 Table**. Modelling parameters for PROMETHEE and GAIA analyse.

|  | **Al** | **As** | **Ba** | **Bi** | **Ca** | **Cd** | **Ce** | **Cr** | **Cu** | **Fe** | **K** | **La** | **Mg** | **Mn** | **Na** | **Pb** | **Sb** | **Sr** | **Ti** | **V** | **W** | **Zn** | **Zr** | Cr^6+^ |
| --- | --- | --- | --- | --- | --- | --- | --- | --- | --- | --- | --- | --- | --- | --- | --- | --- | --- | --- | --- | --- | --- | --- | --- | --- |
| **Unit** | **ng/m^3^** | **ng/m^3^** | **ng/m^3^** | **ng/m^3^** | **ng/m^3^** | **ng/m^3^** | **ng/m^3^** | **ng/m^3^** | **ng/m^3^** | **ng/m^3^** | **ng/m^3^** | **ng/m^3^** | **ng/m^3^** | **ng/m^3^** | **ng/m^3^** | **ng/m^3^** | **ng/m^3^** | **ng/m^3^** | **ng/m^3^** | **ng/m^3^** | **ng/m^3^** | **ng/m^3^** | **ng/m^3^** | ng/m^3^ |
| **Min/Max** | **max** | **max** | **max** | **max** | **max** | **max** | **max** | **max** | **max** | **max** | **max** | **max** | **max** | **max** | **max** | **max** | **max** | **max** | **max** | **max** | **max** | **max** | **max** | max |
| **Weight** | **10** | **600** | **53** | **0** | **0** | **400** | **10** | **10** | **10** | **0** | **0** | **0** | **0** | **10** | **0** | **400** | **10** | **0** | **53** | **53** | **53** | **53** | **0** | 600 |
| KM_2.1-9.0_ | 911.41 | 2.35 | 37.71 | 2.96 | 11247.27 | 0.74 | 1.97 | 9.32 | 12.28 | 2205.11 | 250.71 | 0.99 | 1809.26 | 138.41 | 292.00 | 42.59 | 4.02 | 11.76 | 117.45 | 31.32 | 0.65 | 49.82 | 2.01 | 2.74 |
| WH_2.1-9.0_ | 582.85 | 1.46 | 22.22 | 0.18 | 4525.76 | 0.24 | 1.39 | 4.98 | 12.64 | 1264.36 | 128.74 | 0.74 | 409.71 | 34.54 | 37.10 | 8.12 | 2.81 | 42.88 | 26.33 | 2.40 | 0.98 | 43.77 | 1.01 | 1.59 |
| NJ_2.1-9.0_ | 375.55 | 1.06 | 13.42 | 0.20 | 1731.32 | 0.22 | 1.17 | 3.48 | 6.88 | 1096.83 | 123.40 | 0.89 | 229.53 | 35.31 | 149.86 | 6.38 | 2.21 | 7.26 | 17.77 | 1.90 | 0.24 | 53.00 | 0.78 | 0.25 |
| NB_2.1-9.0_ | 85.56 | 0.27 | 6.13 | 0.03 | 412.28 | 0.05 | 0.38 | 1.66 | 6.41 | 374.97 | 34.06 | 0.22 | 85.61 | 10.78 | 254.62 | 1.31 | 2.46 | 1.65 | 5.30 | 0.71 | 0.16 | 26.30 | 0.28 | 1.07 |
| UN_2.1-9.0_ | 176.50 | 0.19 | 5.74 | 0.03 | 415.86 | 0.04 | 0.42 | 1.70 | 4.91 | 189.11 | 22.35 | 0.18 | 179.82 | 5.16 | 126.55 | 1.39 | 1.52 | 2.70 | 5.28 | 0.29 | 0.14 | 13.92 | 0.41 | 1.01 |
| KM_1.1-2.1_ | 131.94 | 0.50 | 4.38 | 2.60 | 915.44 | 0.25 | 0.26 | 1.04 | 2.19 | 249.31 | 50.95 | 0.12 | 156.42 | 14.53 | 78.15 | 31.24 | 0.81 | 2.19 | 10.19 | 2.04 | 0.10 | 12.89 | 0.57 | 0.57 |
| WH_1.1-2.1_ | 87.12 | 0.98 | 6.18 | 0.18 | 514.53 | 0.28 | 0.27 | 1.44 | 4.35 | 243.25 | 34.34 | 0.17 | 63.59 | 8.76 | 22.24 | 7.10 | 0.90 | 13.24 | 3.40 | 0.55 | 0.20 | 36.44 | 0.35 | 0.35 |
| NJ_1.1-2.1_ | 111.54 | 0.79 | 3.10 | 0.12 | 174.63 | 0.24 | 0.46 | 0.65 | 2.96 | 179.60 | 40.02 | 0.71 | 36.63 | 8.25 | 43.88 | 4.02 | 0.63 | 1.39 | 2.61 | 0.55 | 0.07 | 48.98 | 0.23 | 0.23 |
| NB_1.1-2.1_ | 10.71 | 0.10 | 1.06 | 0.02 | 81.38 | 0.04 | 0.08 | 0.25 | 2.59 | 185.20 | 4.02 | 0.06 | 12.29 | 2.76 | 54.94 | 0.67 | 1.32 | 0.34 | 0.97 | 0.28 | 0.08 | 35.24 | 0.25 | 0.25 |
| UN_1.1-2.1_ | 77.01 | 0.13 | 2.45 | 0.03 | 173.65 | 0.03 | 0.15 | 0.49 | 1.16 | 70.77 | 12.07 | 0.07 | 80.96 | 2.35 | 77.31 | 0.83 | 0.50 | 1.19 | 1.56 | 0.14 | 0.04 | 14.10 | 0.00 | 0.00 |
| KM_1.1_ | 71.29 | 2.55 | 1.42 | 8.62 | 187.73 | 1.23 | 0.09 | 1.60 | 5.87 | 206.82 | 283.81 | 0.04 | 39.55 | 15.63 | 93.56 | 115.75 | 2.72 | 0.67 | 5.55 | 1.18 | 0.09 | 20.74 | 0.26 | 1.36 |
| WH_1.1_ | 49.42 | 5.98 | 3.31 | 1.22 | 153.46 | 1.97 | 0.13 | 2.08 | 28.59 | 283.96 | 224.27 | 0.10 | 17.44 | 18.04 | 55.12 | 40.30 | 5.42 | 4.18 | 2.91 | 1.54 | 0.28 | 89.23 | 0.15 | 1.72 |
| NJ_1.1_ | 77.77 | 4.91 | 3.32 | 1.30 | 92.14 | 3.28 | 0.28 | 2.81 | 14.79 | 276.03 | 1201.44 | 0.45 | 18.03 | 21.84 | 126.00 | 120.72 | 3.19 | 1.18 | 1.93 | 3.98 | 0.33 | 186.84 | 0.11 | 0.96 |
| NB_1.1_ | 118.02 | 1.23 | 2.41 | 0.31 | 21.39 | 0.32 | 0.10 | 3.65 | 10.51 | 338.59 | 84.73 | 0.07 | 12.66 | 10.54 | 51.45 | 8.27 | 2.48 | 0.46 | 1.82 | 5.85 | 0.14 | 73.78 | 0.21 | 1.08 |
| UN_1.1_ | 66.69 | 1.51 | 3.01 | 0.49 | 77.32 | 0.49 | 0.19 | 2.35 | 8.64 | 139.58 | 237.02 | 0.08 | 60.49 | 14.53 | 97.73 | 15.94 | 2.71 | 1.05 | 2.01 | 2.07 | 0.33 | 86.59 | 0.18 | 0.91 |

-The toxicity points from Substance Priority List of Agency for Toxic Substances and Disease Registry 2017 were used for weight coefficients.
