# Supplementary material for: Contamination identification, source apportionment and health risk assessment of trace elements at different fractions of atmospheric particles at iron and steelmaking areas in China
Source: PLoS One. 2020 Apr 2;15(4):e0230983. doi: 10.1371/journal.pone.0230983 (PMC7117772; doi:10.1371/journal.pone.0230983)
Supplement: S2 Table — (DOCX) [file pone.0230983.s003.docx]

**S2 Table**. Bioavailability analysis for trace elements.

|  | **Al** | **As** | **Ba** | **Bi** | **Cd** | **Cr** | **Cu** | **Fe** | **La** | **Mn** | **Pb** | **Sb** | **Sr** | **V** | **Zn** |
| --- | --- | --- | --- | --- | --- | --- | --- | --- | --- | --- | --- | --- | --- | --- | --- |
| KM_2.1-9.0_ | 3.69 | <LOR | 0.25 | 0.02 | <LOR | 0.02 | 0.06 | 4.55 | <LOR | 0.77 | 0.34 | <LOR | 0.06 | 0.19 | 0.22 |
| WH_2.1-9.0_ | 1.48 | <LOR | 0.06 | <LOR | <LOR | 0.04 | 0.02 | 2.10 | <LOR | 0.14 | 0.01 | <LOR | 0.07 | <LOR | 0.15 |
| NJ_2.1-9.0_ | 1.75 | <LOR | 0.10 | <LOR | <LOR | <LOR | 0.08 | 5.55 | <LOR | 0.25 | 0.06 | <LOR | 0.02 | <LOR | 0.66 |
| NB_2.1-9.0_ | 0.14 | <LOR | 0.03 | <LOR | <LOR | <LOR | 0.13 | 0.64 | <LOR | 0.04 | <LOR | <LOR | <LOR | <LOR | 0.35 |
| UN_2.1-9.0_ | 0.19 | <LOR | 0.01 | <LOR | <LOR | <LOR | <LOR | 0.60 | <LOR | 0.04 | <LOR | <LOR | <LOR | <LOR | 0.20 |
| KM_1.1-2.1_ | 0.51 | <LOR | 0.02 | 0.02 | <LOR | <LOR | 0.01 | 0.30 | <LOR | 0.06 | 0.24 | <LOR | <LOR | 0.01 | 0.06 |
| WH_1.1-2.1_ | <LOR | <LOR | 0.02 | <LOR | <LOR | <LOR | 0.02 | 0.09 | <LOR | 0.03 | 0.03 | <LOR | 0.02 | <LOR | 0.40 |
| NJ_1.1-2.1_ | 0.03 | <LOR | 0.02 | <LOR | <LOR | <LOR | 0.03 | 0.33 | <LOR | 0.04 | 0.04 | <LOR | <LOR | <LOR | 0.82 |
| NB_1.1-2.1_ | <LOR | <LOR | <LOR | <LOR | <LOR | <LOR | <LOR | <LOR | <LOR | 0.01 | <LOR | <LOR | <LOR | <LOR | 0.16 |
| UN_1.1-2.1_ | <LOR | <LOR | <LOR | <LOR | <LOR | <LOR | <LOR | <LOR | <LOR | <LOR | <LOR | <LOR | <LOR | <LOR | <LOR |
| KM_1.1_ | 1.18 | <LOR | <LOR | 0.09 | <LOR | <LOR | 0.06 | <LOR | <LOR | 0.03 | 0.93 | <LOR | 0.02 | <LOR | 0.10 |
| WH_1.1_ | 0.23 | 0.25 | 0.02 | 0.03 | 0.01 | <LOR | 0.16 | 0.81 | <LOR | 0.18 | 0.74 | 0.16 | <LOR | <LOR | 2.21 |
| NJ_1.1_ | <LOR | <LOR | <LOR | <LOR | <LOR | <LOR | 0.04 | 0.16 | <LOR | 0.07 | 0.19 | <LOR | <LOR | <LOR | 2.05 |
| NB_1.1_ | 0.17 | <LOR | <LOR | <LOR | <LOR | <LOR | 0.06 | 0.10 | <LOR | 0.04 | 0.04 | <LOR | <LOR | 0.05 | 0.47 |
| UN_1.1_ | 2.60 | <LOR | <LOR | <LOR | <LOR | 0.06 | 0.06 | 0.12 | <LOR | <LOR | <LOR | <LOR | <LOR | <LOR | 0.03 |

-Compared to Table S3 where had 23 trace element concentrations, there were 15 trace elements were analysed for bioavailability. Data for Ca, Ce, K, Mg, Na, Ti, W and Zr are not available.
