# Supplementary material for: Contamination identification, source apportionment and health risk assessment of trace elements at different fractions of atmospheric particles at iron and steelmaking areas in China
Source: PLoS One. 2020 Apr 2;15(4):e0230983. doi: 10.1371/journal.pone.0230983 (PMC7117772; doi:10.1371/journal.pone.0230983)
Supplement: Attachment — Submitted filename: Response to Reviewers.docx [file pone.0230983.s004.docx]

**Response to Reviewers**

***Manuscript ID***: PONE-D-19-32739

***Title***: Contamination identification, source apportionment and health risk assessment of trace elements at different fractions of atmospheric particles at iron and steelmaking areas in China

***Authors***: Xiaoteng Zhou, Vladimir Strezov, Yijiao Jiang, Xiaoxia Yang, Tao Kan and Tim Evans

| **Journal Requirements:** | |
| --- | --- |
| 1. Please ensure that your manuscript meets PLOS ONE's style requirements, including those for file naming. | The formatting and file names were updated according to PLOS ONE’s style requirements. Thank you. |
| 2. In your Methods section, please provide additional location information, including geographic coordinates for the data set if available. | The geographic coordinate for each location was added in the Method section. Please see Lines 119–121. Thank you for your comments to clear the manuscript. |
| 3. We note that Figure 1 in your submission contains map and satellite images which may be copyrighted. All PLOS content is published under the Creative Commons Attribution License (CC BY 4.0), which means that the manuscript, images, and Supporting Information files will be freely available online, and any third party is permitted to access, download, copy, distribute, and use these materials in any way, even commercially, with proper attribution. For these reasons, we cannot publish previously copyrighted maps or satellite images created using proprietary data, such as Google software (Google Maps, Street View, and Earth). | The satellite images included in the Figure 1 were created from the Google Maps. Due to the copyright consideration, these satellite images were removed, but its corresponding information was added as text in the Table 1. |
| 4. PLOS requires an ORCID iD for the corresponding author in Editorial Manager on papers submitted after December 6th, 2016. | The ORCID iD was updated in my PLOS ONE profile. Thank you. |
| 5. Please include captions for your Supporting Information files at the end of your manuscript, and update any in-text citations to match accordingly. | The captions for the supporting information tables were added at the end of the manuscript. Thank you. |

| **Reviewers' comments:** | |
| --- | --- |
| Reviewer #1: The paper presents a comprehensive analysis in source apportionment of trace elements in atmospheric particles collected from four cities, China and estimates the associated health risks with the use of equations of hazard quotient (HQ) and cancer risk (CR), respectively. The paper could be accepted after addressing the following issues.  1. Line 92: Please define climate movement. What is climate movement? | It was changed to ‘atmospheric movement’ to clarify this sentence. Please see Line 99. Thank you. |
| 2. Introductions: PM particles emitted from iron and steel industries posed negative human health not only through toxic trace metals, but also via polycylic aromatic hydrocarbons. Liu et al., Environmental Pollution, 2019, 254, 113046. Liu et al., Science of the Total Environment, 2020, 701, 134844.  Please add a brief introduction with the above references into the updated version of the paper. | Both recommended references are nice work. However, this paper mainly focused on heavy metals rather than PAHs pollution, so there might be far from the topic to have an addition PAHs introduction in the current manuscript, but both papers were cities properly. Please see Line 51. |
| 3. Sampling information: How many samples do you collect for each site during the sampling period? | There were 200 samples collected and analysed in this study (8 samples per site per day). This detail was added on line 157. |
| 4. Data analysis: Please include backward trajectory analysis in the paper in order to testify the emissions from remote sources. | This backward trajectory analysis was added as S2 Figure in this study.  Thank you for the reviewer’s comments to improve this manuscript. |
| Reviewer #2:  I have read the manuscript with great interest. I read it in detail and the truth is that the study is very good. The way the problem, objectives and methodology are contextualize is appropriate. The results are clear and concise. The discussion is detailed and covers the explanation of all the results well. The conclusion highlights the most interesting results of the study. English is clear and all sentences are well stated. I consider that the munuscript is suitable to be published in Plos One without corrections. It is a very interesting work, which can be replicated in other industrialized cities in order to obtain very valuable information for the population and decision makers. | We highly appreciate the reviewer’s positive comments. Thank you again. |
| Reviewer #3: avoid long sentences. | The long sentence was found in Method section, and it has been broken down. Please see Lines 119–122. |
